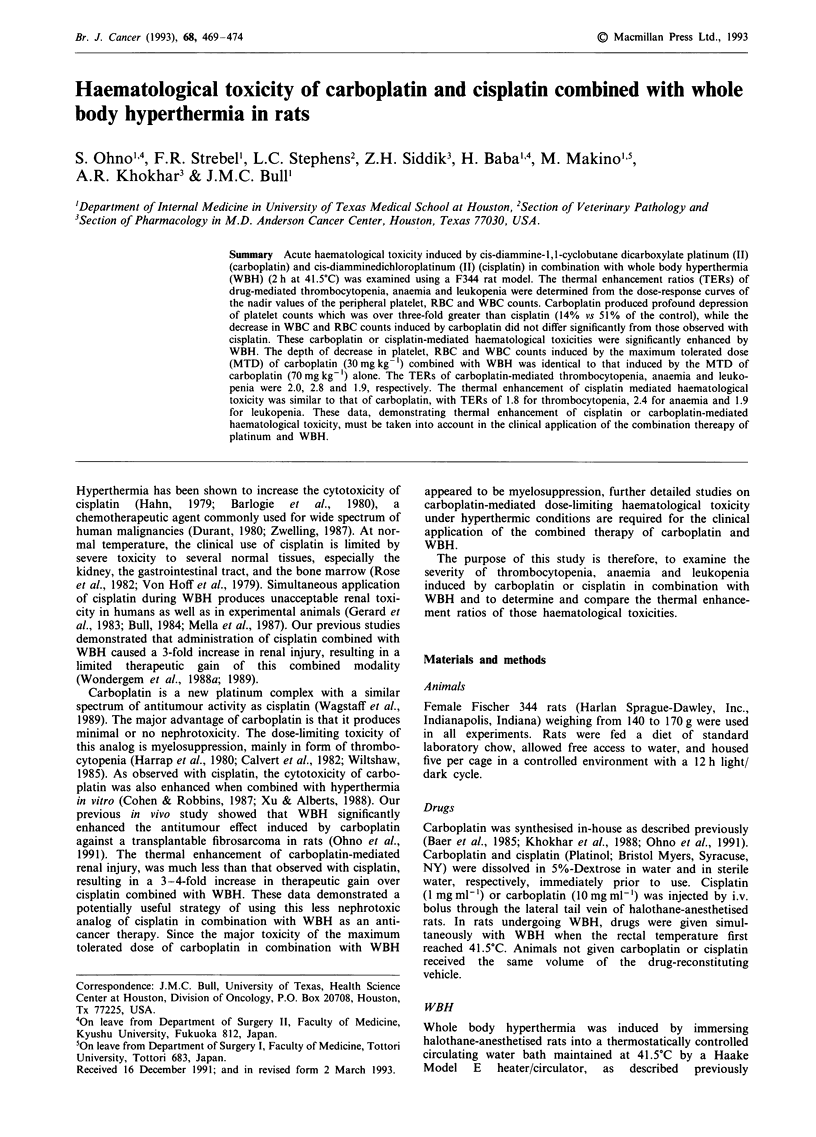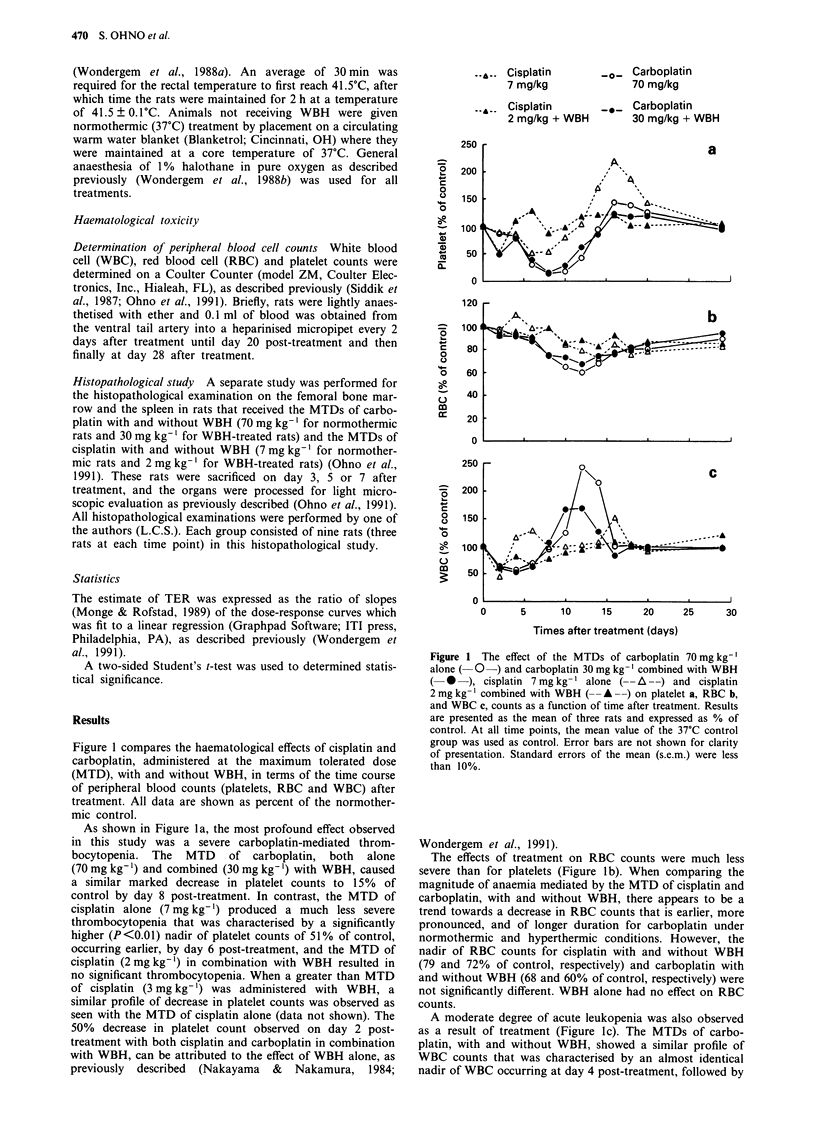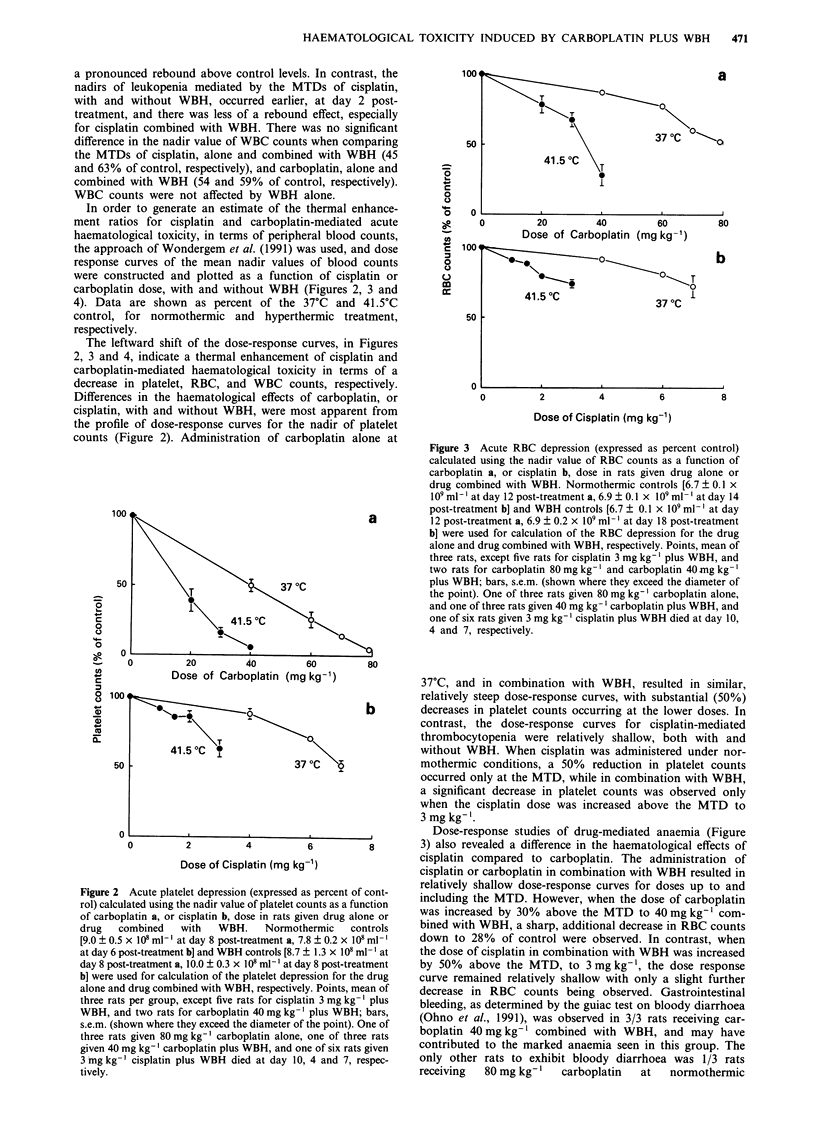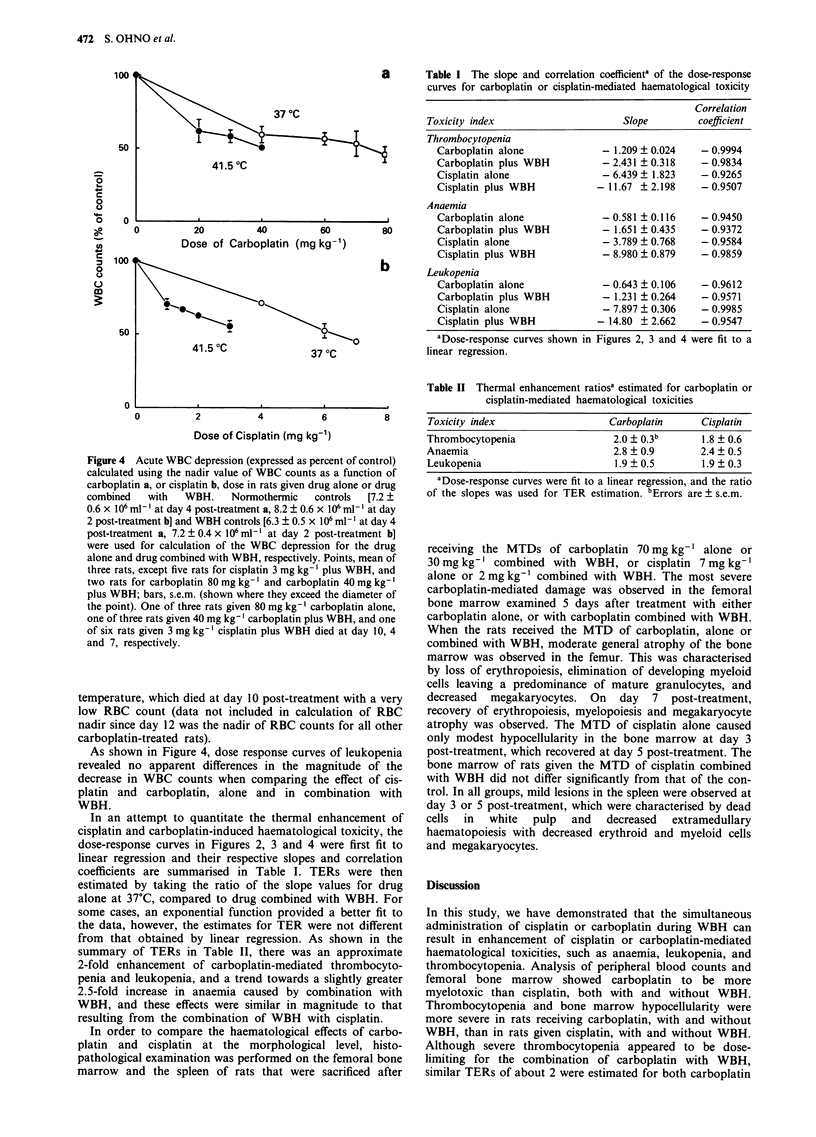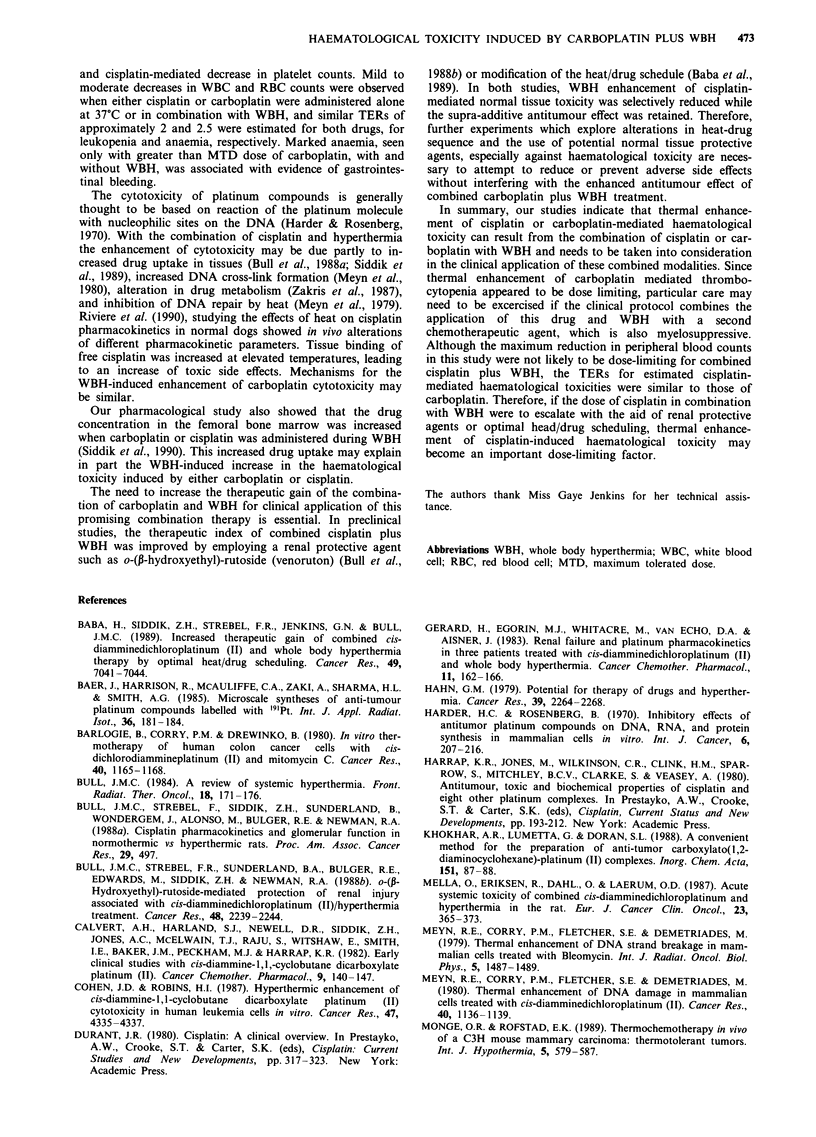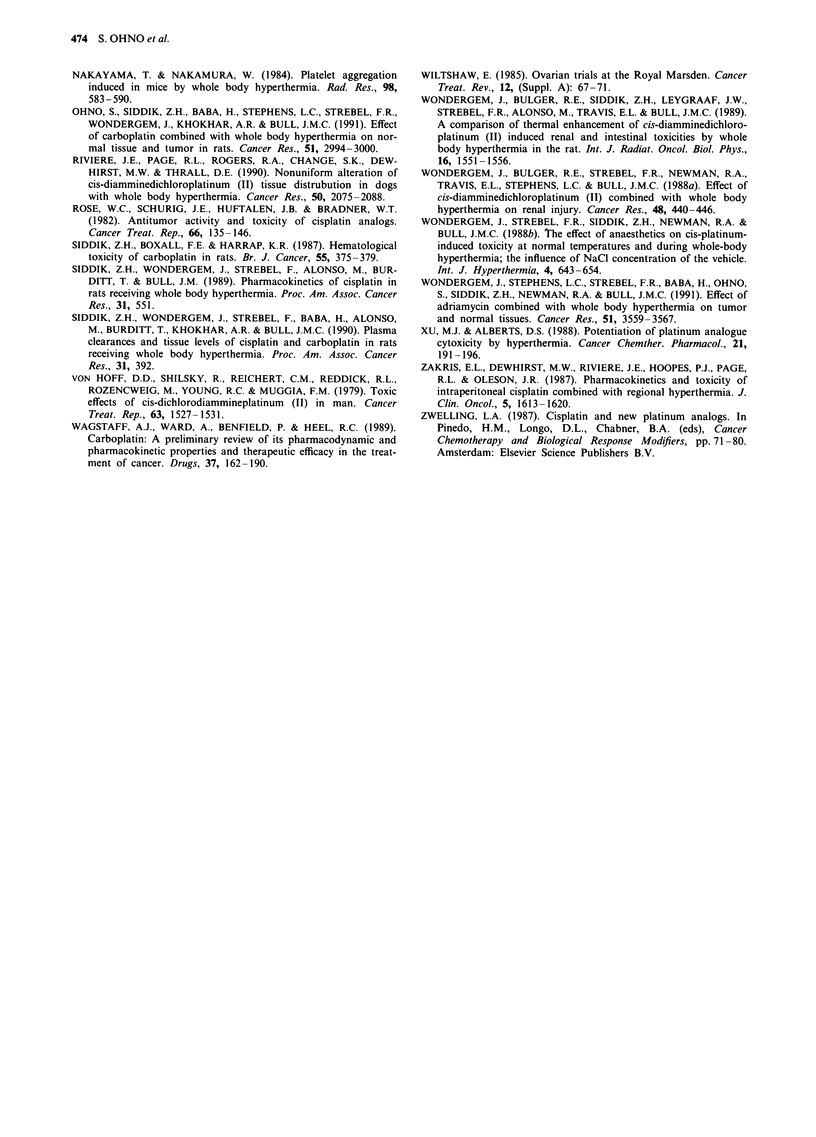# Haematological toxicity of carboplatin and cisplatin combined with whole body hyperthermia in rats.

**DOI:** 10.1038/bjc.1993.372

**Published:** 1993-09

**Authors:** S. Ohno, F. R. Strebel, L. C. Stephens, Z. H. Siddik, H. Baba, M. Makino, A. R. Khokhar, J. M. Bull

**Affiliations:** Department of Internal Medicine in University of Texas Medical School at Houston.

## Abstract

Acute haematological toxicity induced by cis-diammine-1,1-cyclobutane dicarboxylate platinum (II) (carboplatin) and cis-diamminedichloroplatinum (II) (cisplatin) in combination with whole body hyperthermia (WBH) (2 h at 41.5 degrees C) was examined using a F344 rat model. The thermal enhancement ratios (TERs) of drug-mediated thrombocytopenia, anaemia and leukopenia were determined from the dose-response curves of the nadir values of the peripheral platelet, RBC and WBC counts. Carboplatin produced profound depression of platelet counts which was over three-fold greater than cisplatin (14% vs 51% of the control), while the decrease in WBC and RBC counts induced by carboplatin did not differ significantly from those observed with cisplatin. These carboplatin or cisplatin-mediated haematological toxicities were significantly enhanced by WBH. The depth of decrease in platelet, RBC and WBC counts induced by the maximum tolerated dose (MTD) of carboplatin (30 mg kg-1) combined with WBH was identical to that induced by the MTD of carboplatin (70 mg kg-1) alone. The TERs of carboplatin-mediated thrombocytopenia, anaemia and leukopenia were 2.0, 2.8 and 1.9, respectively. The thermal enhancement of cisplatin mediated haematological toxicity was similar to that of carboplatin, with TERs of 1.8 for thrombocytopenia, 2.4 for anaemia and 1.9 for leukopenia. These data, demonstrating thermal enhancement of cisplatin or carboplatin-mediated haematological toxicity, must be taken into account in the clinical application of the combination therapy of platinum and WBH.


Br. J. Cancer (1993), 68, 469-474 © Macmillan Press Ltd., 1993

### Haematological toxicity of carboplatin and cisplatin combined with whole body hyperthermia in rats

S. Ohno[1,4], F.R. Strebel[1], L.C. Stephens[2], Z.H. Siddik[3], H. Baba[1,4], M. Makino[1,5], A.R. Khokhar[3] & J.M.C. Bull[1]

[1]*Department of Internal Medicine in University of Texas Medical School at Houston,* [2]*Section of Veterinary Pathology and* [3]*Section of Pharmacology in M.D. Anderson Cancer Center, Houston, Texas 77030, USA.*

**Summary** Acute haematological toxicity induced by cis-diammine-1,1-cyclobutane dicarboxylate platinum (II) (carboplatin) and cis-diamminedichloroplatinum (II) (cisplatin) in combination with whole body hyperthermia (WBH) (2 h at 41.5°C) was examined using a F344 rat model. The thermal enhancement ratios (TERs) of drug-mediated thrombocytopenia, anaemia and leukopenia were determined from the dose-response curves of the nadir values of the peripheral platelet, RBC and WBC counts. Carboplatin produced profound depression of platelet counts which was over three-fold greater than cisplatin (14% *vs* 51% of the control), while the decrease in WBC and RBC counts induced by carboplatin did not differ significantly from those observed with cisplatin. These carboplatin or cisplatin-mediated haematological toxicities were significantly enhanced by WBH. The depth of decrease in platelet, RBC and WBC counts induced by the maximum tolerated dose (MTD) of carboplatin (30 mg kg$^{-1}$) combined with WBH was identical to that induced by the MTD of carboplatin (70 mg kg$^{-1}$) alone. The TERs of carboplatin-mediated thrombocytopenia, anaemia and leukopenia were 2.0, 2.8 and 1.9, respectively. The thermal enhancement of cisplatin mediated haematological toxicity was similar to that of carboplatin, with TERs of 1.8 for thrombocytopenia, 2.4 for anaemia and 1.9 for leukopenia. These data, demonstrating thermal enhancement of cisplatin or carboplatin-mediated haematological toxicity, must be taken into account in the clinical application of the combination thereapy of platinum and WBH.

Hyperthermia has been shown to increase the cytotoxicity of cisplatin (Hahn, 1979; Barlogie *et al.*, 1980), a chemotherapeutic agent commonly used for wide spectrum of human malignancies (Durant, 1980; Zwelling, 1987). At normal temperature, the clinical use of cisplatin is limited by severe toxicity to several normal tissues, especially the kidney, the gastrointestinal tract, and the bone marrow (Rose *et al.*, 1982; Von Hoff *et al.*, 1979). Simultaneous application of cisplatin during WBH produces unacceptable renal toxicity in humans as well as in experimental animals (Gerard *et al.*, 1983; Bull, 1984; Mella *et al.*, 1987). Our previous studies demonstrated that administration of cisplatin combined with WBH caused a 3-fold increase in renal injury, resulting in a limited therapeutic gain of this combined modality (Wondergem *et al.*, 1988*a*; 1989).

Carboplatin is a new platinum complex with a similar spectrum of antitumour activity as cisplatin (Wagstaff *et al.*, 1989). The major advantage of carboplatin is that it produces minimal or no nephrotoxicity. The dose-limiting toxicity of this analog is myelosuppression, mainly in form of thrombocytopenia (Harrap *et al.*, 1980; Calvert *et al.*, 1982; Wiltshaw, 1985). As observed with cisplatin, the cytotoxicity of carboplatin was also enhanced when combined with hyperthermia *in vitro* (Cohen & Robbins, 1987; Xu & Alberts, 1988). Our previous *in vivo* study showed that WBH significantly enhanced the antitumour effect induced by carboplatin against a transplantable fibrosarcoma in rats (Ohno *et al.*, 1991). The thermal enhancement of carboplatin-mediated renal injury, was much less than that observed with cisplatin, resulting in a 3-4-fold increase in therapeutic gain over cisplatin combined with WBH. These data demonstrated a potentially useful strategy of using this less nephrotoxic analog of cisplatin in combination with WBH as an anticancer therapy. Since the major toxicity of the maximum tolerated dose of carboplatin in combination with WBH appeared to be myelosuppression, further detailed studies on carboplatin-mediated dose-limiting haematological toxicity under hyperthermic conditions are required for the clinical application of the combined therapy of carboplatin and WBH.

The purpose of this study is therefore, to examine the severity of thrombocytopenia, anaemia and leukopenia induced by carboplatin or cisplatin in combination with WBH and to determine and compare the thermal enhancement ratios of those haematological toxicities.

### Materials and methods

### *Animals*

Female Fischer 344 rats (Harlan Sprague-Dawley, Inc., Indianapolis, Indiana) weighing from 140 to 170 g were used in all experiments. Rats were fed a diet of standard laboratory chow, allowed free access to water, and housed five per cage in a controlled environment with a 12 h light/dark cycle.

### *Drugs*

Carboplatin was synthesised in-house as described previously (Baer *et al.*, 1985; Khokhar *et al.*, 1988; Ohno *et al.*, 1991). Carboplatin and cisplatin (Platinol; Bristol Myers, Syracuse, NY) were dissolved in 5%-Dextrose in water and in sterile water, respectively, immediately prior to use. Cisplatin (1 mg ml$^{-1}$) or carboplatin (10 mg ml$^{-1}$) was injected by i.v. bolus through the lateral tail vein of halothane-anesthetised rats. In rats undergoing WBH, drugs were given simultaneously with WBH when the rectal temperature first reached 41.5°C. Animals not given carboplatin or cisplatin received the same volume of the drug-reconstituting vehicle.

### *WBH*

Whole body hyperthermia was induced by immersing halothane-anesthetised rats into a thermostatically controlled circulating water bath maintained at 41.5°C by a Haake Model E heater/circulator, as described previously

Correspondence: J.M.C. Bull, University of Texas, Health Science Center at Houston, Division of Oncology, P.O. Box 20708, Houston, Tx 77225, USA.
[4]On leave from Department of Surgery II, Faculty of Medicine, Kyushu University, Fukuoka 812, Japan.
[5]On leave from Department of Surgery I, Faculty of Medicine, Tottori University, Tottori 683, Japan.
Received 16 December 1991; and in revised form 2 March 1993.

(Wondergem et al., 1988a). An average of 30 min was required for the rectal temperature to first reach 41.5°C, after which time the rats were maintained for 2 h at a temperature of 41.5 ± 0.1°C. Animals not receiving WBH were given normothermic (37°C) treatment by placement on a circulating warm water blanket (Blanketrol; Cincinnati, OH) where they were maintained at a core temperature of 37°C. General anaesthesia of 1% halothane in pure oxygen as described previously (Wondergem et al., 1988b) was used for all treatments.

*Haematological toxicity*

*Determination of peripheral blood cell counts* White blood cell (WBC), red blood cell (RBC) and platelet counts were determined on a Coulter Counter (model ZM, Coulter Electronics, Inc., Hialeah, FL), as described previously (Siddik et al., 1987; Ohno et al., 1991). Briefly, rats were lightly anaesthetised with ether and 0.1 ml of blood was obtained from the ventral tail artery into a heparinised micropipet every 2 days after treatment until day 20 post-treatment and then finally at day 28 after treatment.

*Histopathological study* A separate study was performed for the histopathological examination on the femoral bone marrow and the spleen in rats that received the MTDs of carboplatin with and without WBH (70 mg $kg^{-1}$ for normothermic rats and 30 mg $kg^{-1}$ for WBH-treated rats) and the MTDs of cisplatin with and without WBH (7 mg $kg^{-1}$ for normothermic rats and 2 mg $kg^{-1}$ for WBH-treated rats) (Ohno et al., 1991). These rats were sacrificed on day 3, 5 or 7 after treatment, and the organs were processed for light microscopic evaluation as previously described (Ohno et al., 1991). All histopathological examinations were performed by one of the authors (L.C.S.). Each group consisted of nine rats (three rats at each time point) in this histopathological study.

*Statistics*

The estimate of TER was expressed as the ratio of slopes (Monge & Rofstad, 1989) of the dose-response curves which was fit to a linear regression (Graphpad Software; ITI press, Philadelphia, PA), as described previously (Wondergem et al., 1991).

A two-sided Student's t-test was used to determined statistical significance.

### Results

Figure 1 compares the haematological effects of cisplatin and carboplatin, administered at the maximum tolerated dose (MTD), with and without WBH, in terms of the time course of peripheral blood counts (platelets, RBC and WBC) after treatment. All data are shown as percent of the normothermic control.

As shown in Figure 1a, the most profound effect observed in this study was a severe carboplatin-mediated thrombocytopenia. The MTD of carboplatin, both alone (70 mg $kg^{-1}$) and combined (30 mg $kg^{-1}$) with WBH, caused a similar marked decrease in platelet counts to 15% of control by day 8 post-treatment. In contrast, the MTD of cisplatin alone (7 mg $kg^{-1}$) produced a much less severe thrombocytopenia that was characterised by a significantly higher ($P<0.01$) nadir of platelet counts of 51% of control, occurring earlier, by day 6 post-treatment, and the MTD of cisplatin (2 mg $kg^{-1}$) in combination with WBH resulted in no significant thrombocytopenia. When a greater than MTD of cisplatin (3 mg $kg^{-1}$) was administered with WBH, a similar profile of decrease in platelet counts was observed as seen with the MTD of cisplatin alone (data not shown). The 50% decrease in platelet count observed on day 2 post-treatment with both cisplatin and carboplatin in combination with WBH, can be attributed to the effect of WBH alone, as previously described (Nakayama & Nakamura, 1984; Wondergem et al., 1991).

**Figure 1** The effect of the MTDs of carboplatin 70 mg $kg^{-1}$ alone (—O—) and carboplatin 30 mg $kg^{-1}$ combined with WBH (—●—), cisplatin 7 mg $kg^{-1}$ alone (--Δ--) and cisplatin 2 mg $kg^{-1}$ combined with WBH (--▲--) on platelet **a**, RBC **b**, and WBC **c**, counts as a function of time after treatment. Results are presented as the mean of three rats and expressed as % of control. At all time points, the mean value of the 37°C control group was used as control. Error bars are not shown for clarity of presentation. Standard errors of the mean (s.e.m.) were less than 10%.

The effects of treatment on RBC counts were much less severe than for platelets (Figure 1b). When comparing the magnitude of anaemia mediated by the MTD of cisplatin and carboplatin, with and without WBH, there appears to be a trend towards a decrease in RBC counts that is earlier, more pronounced, and of longer duration for carboplatin under normothermic and hyperthermic conditions. However, the nadir of RBC counts for cisplatin with and without WBH (79 and 72% of control, respectively) and carboplatin with and without WBH (68 and 60% of control, respectively) were not significantly different. WBH alone had no effect on RBC counts.

A moderate degree of acute leukopenia was also observed as a result of treatment (Figure 1c). The MTDs of carboplatin, with and without WBH, showed a similar profile of WBC counts that was characterised by an almost identical nadir of WBC occurring at day 4 post-treatment, followed by a pronounced rebound above control levels. In contrast, the nadirs of leukopenia mediated by the MTDs of cisplatin, with and without WBH, occurred earlier, at day 2 post-treatment, and there was less of a rebound effect, especially for cisplatin combined with WBH. There was no significant difference in the nadir value of WBC counts when comparing the MTDs of cisplatin, alone and combined with WBH (45 and 63% of control, respectively), and carboplatin, alone and combined with WBH (54 and 59% of control, respectively). WBC counts were not affected by WBH alone.

In order to generate an estimate of the thermal enhancement ratios for cisplatin and carboplatin-mediated acute haematological toxicity, in terms of peripheral blood counts, the approach of Wondergem *et al.* (1991) was used, and dose response curves of the mean nadir values of blood counts were constructed and plotted as a function of cisplatin or carboplatin dose, with and without WBH (Figures 2, 3 and 4). Data are shown as percent of the 37°C and 41.5°C control, for normothermic and hyperthermic treatment, respectively.

The leftward shift of the dose-response curves, in Figures 2, 3 and 4, indicate a thermal enhancement of cisplatin and carboplatin-mediated haematological toxicity in terms of a decrease in platelet, RBC, and WBC counts, respectively. Differences in the haematological effects of carboplatin, or cisplatin, with and without WBH, were most apparent from the profile of dose-response curves for the nadir of platelet counts (Figure 2). Administration of carboplatin alone at

**Figure 2** Acute platelet depression (expressed as percent of control) calculated using the nadir value of platelet counts as a function of carboplatin **a**, or cisplatin **b**, dose in rats given drug alone or drug combined with WBH. Normothermic controls [$9.0 \pm 0.5 \times 10^8$ ml$^{-1}$ at day 8 post-treatment **a**, $7.8 \pm 0.2 \times 10^8$ ml$^{-1}$ at day 6 post-treatment **b**] and WBH controls [$8.7 \pm 1.3 \times 10^8$ ml$^{-1}$ at day 8 post-treatment **a**, $10.0 \pm 0.3 \times 10^8$ ml$^{-1}$ at day 8 post-treatment **b**] were used for calculation of the platelet depression for the drug alone and drug combined with WBH, respectively. Points, mean of three rats per group, except five rats for cisplatin 3 mg kg$^{-1}$ plus WBH, and two rats for carboplatin 40 mg kg$^{-1}$ plus WBH; bars, s.e.m. (shown where they exceed the diameter of the point). One of three rats given 80 mg kg$^{-1}$ carboplatin alone, one of three rats given 40 mg kg$^{-1}$ carboplatin plus WBH, and one of six rats given 3 mg kg$^{-1}$ cisplatin plus WBH died at day 10, 4 and 7, respectively.

**Figure 3** Acute RBC depression (expressed as percent control) calculated using the nadir value of RBC counts as a function of carboplatin **a**, or cisplatin **b**, dose in rats given drug alone or drug combined with WBH. Normothermic controls [$6.7 \pm 0.1 \times 10^9$ ml$^{-1}$ at day 12 post-treatment **a**, $6.9 \pm 0.1 \times 10^9$ ml$^{-1}$ at day 14 post-treatment **b**] and WBH controls [$6.7 \pm 0.1 \times 10^9$ ml$^{-1}$ at day 12 post-treatment **a**, $6.9 \pm 0.2 \times 10^9$ ml$^{-1}$ at day 18 post-treatment **b**] were used for calculation of the RBC depression for the drug alone and drug combined with WBH, respectively. Points, mean of three rats, except five rats for cisplatin 3 mg kg$^{-1}$ plus WBH, and two rats for carboplatin 80 mg kg$^{-1}$ and carboplatin 40 mg kg$^{-1}$ plus WBH; bars, s.e.m. (shown where they exceed the diameter of the point). One of three rats given 80 mg kg$^{-1}$ carboplatin alone, and one of three rats given 40 mg kg$^{-1}$ carboplatin plus WBH, and one of six rats given 3 mg kg$^{-1}$ cisplatin plus WBH died at day 10, 4 and 7, respectively.

37°C, and in combination with WBH, resulted in similar, relatively steep dose-response curves, with substantial (50%) decreases in platelet counts occurring at the lower doses. In contrast, the dose-response curves for cisplatin-mediated thrombocytopenia were relatively shallow, both with and without WBH. When cisplatin was administered under normothermic conditions, a 50% reduction in platelet counts occurred only at the MTD, while in combination with WBH, a significant decrease in platelet counts was observed only when the cisplatin dose was increased above the MTD to 3 mg kg$^{-1}$.

Dose-response studies of drug-mediated anaemia (Figure 3) also revealed a difference in the haematological effects of cisplatin compared to carboplatin. The administration of cisplatin or carboplatin in combination with WBH resulted in relatively shallow dose-response curves for doses up to and including the MTD. However, when the dose of carboplatin was increased by 30% above the MTD to 40 mg kg$^{-1}$ combined with WBH, a sharp, additional decrease in RBC counts down to 28% of control were observed. In contrast, when the dose of cisplatin in combination with WBH was increased by 50% above the MTD, to 3 mg kg$^{-1}$, the dose response curve remained relatively shallow with only a slight further decrease in RBC counts being observed. Gastrointestinal bleeding, as determined by the guiac test on bloody diarrhoea (Ohno *et al.*, 1991), was observed in 3/3 rats receiving carboplatin 40 mg kg$^{-1}$ combined with WBH, and may have contributed to the marked anaemia seen in this group. The only other rats to exhibit bloody diarrhoea was 1/3 rats receiving 80 mg kg$^{-1}$ carboplatin at normothermic

**Figure 4** Acute WBC depression (expressed as percent of control) calculated using the nadir value of WBC counts as a function of carboplatin **a**, or cisplatin **b**, dose in rats given drug alone or drug combined with WBH. Normothermic controls [7.2 ± 0.6 × 10$^6$ ml$^{-1}$ at day 4 post-treatment **a**, 8.2 ± 0.6 × 10$^6$ ml$^{-1}$ at day 2 post-treatment **b**] and WBH controls [6.3 ± 0.5 × 10$^6$ ml$^{-1}$ at day 4 post-treatment **a**, 7.2 ± 0.4 × 10$^6$ ml$^{-1}$ at day 2 post-treatment **b**] were used for calculation of the WBC depression for the drug alone and drug combined with WBH, respectively. Points, mean of three rats, except five rats for cisplatin 3 mg kg$^{-1}$ plus WBH, and two rats for carboplatin 80 mg kg$^{-1}$ and carboplatin 40 mg kg$^{-1}$ plus WBH; bars, s.e.m. (shown where they exceed the diameter of the point). One of three rats given 80 mg kg$^{-1}$ carboplatin alone, one of three rats given 40 mg kg$^{-1}$ carboplatin plus WBH, and one of six rats given 3 mg kg$^{-1}$ cisplatin plus WBH died at day 10, 4 and 7, respectively.

temperature, which died at day 10 post-treatment with a very low RBC count (data not included in calculation of RBC nadir since day 12 was the nadir of RBC counts for all other carboplatin-treated rats).

As shown in Figure 4, dose response curves of leukopenia revealed no apparent differences in the magnitude of the decrease in WBC counts when comparing the effect of cisplatin and carboplatin, alone and in combination with WBH.

In an attempt to quantitate the thermal enhancement of cisplatin and carboplatin-induced haematological toxicity, the dose-response curves in Figures 2, 3 and 4 were first fit to linear regression and their respective slopes and correlation coefficients are summarised in Table I. TERs were then estimated by taking the ratio of the slope values for drug alone at 37°C, compared to drug combined with WBH. For some cases, an exponential function provided a better fit to the data, however, the estimates for TER were not different from that obtained by linear regression. As shown in the summary of TERs in Table II, there was an approximate 2-fold enhancement of carboplatin-mediated thrombocytopenia and leukopenia, and a trend towards a slightly greater 2.5-fold increase in anaemia caused by combination with WBH, and these effects were similar in magnitude to that resulting from the combination of WBH with cisplatin.

In order to compare the haematological effects of carboplatin and cisplatin at the morphological level, histopathological examination was performed on the femoral bone marrow and the spleen of rats that were sacrificed after

**Table I** The slope and correlation coefficient[a] of the dose-response curves for carboplatin or cisplatin-mediated haematological toxicity

| *Toxicity index* | *Slope* | *Correlation coefficient* |
|---|---|---|
| *Thrombocytopenia* | | |
| Carboplatin alone | – 1.209 ± 0.024 | – 0.9994 |
| Carboplatin plus WBH | – 2.431 ± 0.318 | – 0.9834 |
| Cisplatin alone | – 6.439 ± 1.823 | – 0.9265 |
| Cisplatin plus WBH | – 11.67 ± 2.198 | – 0.9507 |
| *Anaemia* | | |
| Carboplatin alone | – 0.581 ± 0.116 | – 0.9450 |
| Carboplatin plus WBH | – 1.651 ± 0.435 | – 0.9372 |
| Cisplatin alone | – 3.789 ± 0.768 | – 0.9584 |
| Cisplatin plus WBH | – 8.980 ± 0.879 | – 0.9859 |
| *Leukopenia* | | |
| Carboplatin alone | – 0.643 ± 0.106 | – 0.9612 |
| Carboplatin plus WBH | – 1.231 ± 0.264 | – 0.9571 |
| Cisplatin alone | – 7.897 ± 0.306 | – 0.9985 |
| Cisplatin plus WBH | – 14.80 ± 2.662 | – 0.9547 |

[a]Dose-response curves shown in Figures 2, 3 and 4 were fit to a linear regression.

**Table II** Thermal enhancement ratios[a] estimated for carboplatin or cisplatin-mediated haematological toxicities

| *Toxicity index* | *Carboplatin* | *Cisplatin* |
|---|---|---|
| Thrombocytopenia | 2.0 ± 0.3[b] | 1.8 ± 0.6 |
| Anaemia | 2.8 ± 0.9 | 2.4 ± 0.5 |
| Leukopenia | 1.9 ± 0.5 | 1.9 ± 0.3 |

[a]Dose-response curves were fit to a linear regression, and the ratio of the slopes was used for TER estimation. [b]Errors are ± s.e.m.

receiving the MTDs of carboplatin 70 mg kg$^{-1}$ alone or 30 mg kg$^{-1}$ combined with WBH, or cisplatin 7 mg kg$^{-1}$ alone or 2 mg kg$^{-1}$ combined with WBH. The most severe carboplatin-mediated damage was observed in the femoral bone marrow examined 5 days after treatment with either carboplatin alone, or with carboplatin combined with WBH. When the rats received the MTD of carboplatin, alone or combined with WBH, moderate general atrophy of the bone marrow was observed in the femur. This was characterised by loss of erythropoiesis, elimination of developing myeloid cells leaving a predominance of mature granulocytes, and decreased megakaryocytes. On day 7 post-treatment, recovery of erythropoiesis, myelopoiesis and megakaryocyte atrophy was observed. The MTD of cisplatin alone caused only modest hypocellularity in the bone marrow at day 3 post-treatment, which recovered at day 5 post-treatment. The bone marrow of rats given the MTD of cisplatin combined with WBH did not differ significantly from that of the control. In all groups, mild lesions in the spleen were observed at day 3 or 5 post-treatment, which were characterised by dead cells in white pulp and decreased extramedullary haematopoiesis with decreased erythroid and myeloid cells and megakaryocytes.

### Discussion

In this study, we have demonstrated that the simultaneous administration of cisplatin or carboplatin during WBH can result in enhancement of cisplatin or carboplatin-mediated haematological toxicities, such as anaemia, leukopenia, and thrombocytopenia. Analysis of peripheral blood counts and femoral bone marrow showed carboplatin to be more myelotoxic than cisplatin, both with and without WBH. Thrombocytopenia and bone marrow hypocellularity were more severe in rats receiving carboplatin, with and without WBH, than in rats given cisplatin, with and without WBH. Although severe thrombocytopenia appeared to be dose-limiting for the combination of carboplatin with WBH, similar TERs of about 2 were estimated for both carboplatin and cisplatin-mediated decrease in platelet counts. Mild to moderate decreases in WBC and RBC counts were observed when either cisplatin or carboplatin were administered alone at 37°C or in combination with WBH, and similar TERs of approximately 2 and 2.5 were estimated for both drugs, for leukopenia and anaemia, respectively. Marked anaemia, seen only with greater than MTD dose of carboplatin, with and without WBH, was associated with evidence of gastrointestinal bleeding.

The cytotoxicity of platinum compounds is generally thought to be based on reaction of the platinum molecule with nucleophilic sites on the DNA (Harder & Rosenberg, 1970). With the combination of cisplatin and hyperthermia the enhancement of cytotoxicity may be due partly to increased drug uptake in tissues (Bull *et al.*, 1988*a*; Siddik *et al.*, 1989), increased DNA cross-link formation (Meyn *et al.*, 1980), alteration in drug metabolism (Zakris *et al.*, 1987), and inhibition of DNA repair by heat (Meyn *et al.*, 1979). Riviere *et al.* (1990), studying the effects of heat on cisplatin pharmacokinetics in normal dogs showed *in vivo* alterations of different pharmacokinetic parameters. Tissue binding of free cisplatin was increased at elevated temperatures, leading to an increase of toxic side effects. Mechanisms for the WBH-induced enhancement of carboplatin cytotoxicity may be similar.

Our pharmacological study also showed that the drug concentration in the femoral bone marrow was increased when carboplatin or cisplatin was administered during WBH (Siddik *et al.*, 1990). This increased drug uptake may explain in part the WBH-induced increase in the haematological toxicity induced by either carboplatin or cisplatin.

The need to increase the therapeutic gain of the combination of carboplatin and WBH for clinical application of this promising combination therapy is essential. In preclinical studies, the therapeutic index of combined cisplatin plus WBH was improved by employing a renal protective agent such as *o*-(β-hydroxyethyl)-rutoside (venoruton) (Bull *et al.*, 1988*b*) or modification of the heat/drug schedule (Baba *et al.*, 1989). In both studies, WBH enhancement of cisplatin-mediated normal tissue toxicity was selectively reduced while the supra-additive antitumour effect was retained. Therefore, further experiments which explore alterations in heat-drug sequence and the use of potential normal tissue protective agents, especially against haematological toxicity are necessary to attempt to reduce or prevent adverse side effects without interfering with the enhanced antitumour effect of combined carboplatin plus WBH treatment.

In summary, our studies indicate that thermal enhancement of cisplatin or carboplatin-mediated haematological toxicity can result from the combination of cisplatin or carboplatin with WBH and needs to be taken into consideration in the clinical application of these combined modalities. Since thermal enhancement of carboplatin mediated thrombocytopenia appeared to be dose limiting, particular care may need to be excercised if the clinical protocol combines the application of this drug and WBH with a second chemotherapeutic agent, which is also myelosuppressive. Although the maximum reduction in peripheral blood counts in this study were not likely to be dose-limiting for combined cisplatin plus WBH, the TERs for estimated cisplatin-mediated haematological toxicities were similar to those of carboplatin. Therefore, if the dose of cisplatin in combination with WBH were to escalate with the aid of renal protective agents or optimal head/drug scheduling, thermal enhancement of cisplatin-induced haematological toxicity may become an important dose-limiting factor.

The authors thank Miss Gaye Jenkins for her technical assistance.

**Abbreviations** WBH, whole body hyperthermia; WBC, white blood cell; RBC, red blood cell; MTD, maximum tolerated dose.

### References

BABA, H., SIDDIK, Z.H., STREBEL, F.R., JENKINS, G.N. & BULL, J.M.C. (1989). Increased therapeutic gain of combined *cis*-diamminedichloroplatinum (II) and whole body hyperthermia therapy by optimal heat/drug scheduling. *Cancer Res.*, **49**, 7041–7044.

BAER, J., HARRISON, R., MCAULIFFE, C.A., ZAKI, A., SHARMA, H.L. & SMITH, A.G. (1985). Microscale syntheses of anti-tumour platinum compounds labelled with $^{191}$Pt. *Int. J. Appl. Radiat. Isot.*, **36**, 181–184.

BARLOGIE, B., CORRY, P.M. & DREWINKO, B. (1980). *In vitro* thermotherapy of human colon cancer cells with *cis*-dichlorodiammineplatinum (II) and mitomycin C. *Cancer Res.*, **40**, 1165–1168.

BULL, J.M.C. (1984). A review of systemic hyperthermia. *Front. Radiat. Ther. Oncol.*, **18**, 171–176.

BULL, J.M.C., STREBEL, F., SIDDIK, Z.H., SUNDERLAND, B., WONDERGEM, J., ALONSO, M., BULGER, R.E. & NEWMAN, R.A. (1988*a*). Cisplatin pharmacokinetics and glomerular function in normothermic *vs* hyperthermic rats. *Proc. Am. Assoc. Cancer Res.*, **29**, 497.

BULL, J.M.C., STREBEL, F.R., SUNDERLAND, B.A., BULGER, R.E., EDWARDS, M., SIDDIK, Z.H. & NEWMAN, R.A. (1988*b*). *o*-(β-Hydroxyethyl)-rutoside-mediated protection of renal injury associated with *cis*-diamminedichloroplatinum (II)/hyperthermia treatment. *Cancer Res.*, **48**, 2239–2244.

CALVERT, A.H., HARLAND, S.J., NEWELL, D.R., SIDDIK, Z.H., JONES, A.C., MCELWAIN, T.J., RAJU, S., WITSHAW, E., SMITH, I.E., BAKER, J.M., PECKHAM, M.J. & HARRAP, K.R. (1982). Early clinical studies with *cis*-diammine-1,1,-cyclobutane dicarboxylate platinum (II). *Cancer Chemother. Pharmacol.*, **9**, 140–147.

COHEN, J.D. & ROBINS, H.I. (1987). Hyperthermic enhancement of *cis*-diammine-1,1-cyclobutane dicarboxylate platinum (II) cytotoxicity in human leukemia cells *in vitro*. *Cancer Res.*, **47**, 4335–4337.

DURANT, J.R. (1980). Cisplatin: A clinical overview. In Prestayko, A.W., Crooke, S.T. & Carter, S.K. (eds), *Cisplatin: Current Studies and New Developments*, pp. 317–323. New York: Academic Press.

GERARD, H., EGORIN, M.J., WHITACRE, M., VAN ECHO, D.A. & AISNER, J. (1983). Renal failure and platinum pharmacokinetics in three patients treated with *cis*-diamminedichloroplatinum (II) and whole body hyperthermia. *Cancer Chemother. Pharmacol.*, **11**, 162–166.

HAHN, G.M. (1979). Potential for therapy of drugs and hyperthermia. *Cancer Res.*, **39**, 2264–2268.

HARDER, H.C. & ROSENBERG, B. (1970). Inhibitory effects of antitumor platinum compounds on DNA, RNA, and protein synthesis in mammalian cells *in vitro*. *Int. J. Cancer*, **6**, 207–216.

HARRAP, K.R., JONES, M., WILKINSON, C.R., CLINK, H.M., SPARROW, S., MITCHLEY, B.C.V., CLARKE, S. & VEASEY, A. (1980). Antitumour, toxic and biochemical properties of cisplatin and eight other platinum complexes. In Prestayko, A.W., Crooke, S.T. & Carter, S.K. (eds), *Cisplatin, Current Status and New Developments*, pp. 193-212. New York: Academic Press.

KHOKHAR, A.R., LUMETTA, G. & DORAN, S.L. (1988). A convenient method for the preparation of anti-tumor carboxylato(1,2-diaminocyclohexane)-platinum (II) complexes. *Inorg. Chem. Acta*, **151**, 87–88.

MELLA, O., ERIKSEN, R., DAHL., O. & LAERUM, O.D. (1987). Acute systemic toxicity of combined *cis*-diamminedichloroplatinum and hyperthermia in the rat. *Eur. J. Cancer Clin. Oncol.*, **23**, 365–373.

MEYN, R.E., CORRY, P.M., FLETCHER, S.E. & DEMETRIADES, M. (1979). Thermal enhancement of DNA strand breakage in mammalian cells treated with Bleomycin. *Int. J. Radiat. Oncol. Biol. Phys.*, **5**, 1487–1489.

MEYN, R.E., CORRY, P.M., FLETCHER, S.E. & DEMETRIADES, M. (1980). Thermal enhancement of DNA damage in mammalian cells treated with *cis*-diamminedichloroplatinum (II). *Cancer Res.*, **40**, 1136–1139.

MONGE, O.R. & ROFSTAD, E.K. (1989). Thermochemotherapy *in vivo* of a C3H mouse mammary carcinoma: thermotolerant tumors. *Int. J. Hypothermia*, **5**, 579–587.

NAKAYAMA, T. & NAKAMURA, W. (1984). Platelet aggregation induced in mice by whole body hyperthermia. *Rad. Res.*, **98,** 583–590.

OHNO, S., SIDDIK, Z.H., BABA, H., STEPHENS, L.C., STREBEL, F.R., WONDERGEM, J., KHOKHAR, A.R. & BULL, J.M.C. (1991). Effect of carboplatin combined with whole body hyperthermia on normal tissue and tumor in rats. *Cancer Res.*, **51,** 2994–3000.

RIVIERE, J.E., PAGE, R.L., ROGERS, R.A., CHANGE, S.K., DEWHIRST, M.W. & THRALL, D.E. (1990). Nonuniform alteration of cis-diamminedichloroplatinum (II) tissue distrubution in dogs with whole body hyperthermia. *Cancer Res.*, **50,** 2075–2088.

ROSE, W.C., SCHURIG, J.E., HUFTALEN, J.B. & BRADNER, W.T. (1982). Antitumor activity and toxicity of cisplatin analogs. *Cancer Treat. Rep.*, **66,** 135–146.

SIDDIK, Z.H., BOXALL, F.E. & HARRAP, K.R. (1987). Hematological toxicity of carboplatin in rats. *Br. J. Cancer*, **55,** 375–379.

SIDDIK, Z.H., WONDERGEM, J., STREBEL, F., ALONSO, M., BURDITT, T. & BULL, J.M. (1989). Pharmacokinetics of cisplatin in rats receiving whole body hyperthermia. *Proc. Am. Assoc. Cancer Res.*, **31,** 551.

SIDDIK, Z.H., WONDERGEM, J., STREBEL, F., BABA, H., ALONSO, M., BURDITT, T., KHOKHAR, A.R. & BULL, J.M.C. (1990). Plasma clearances and tissue levels of cisplatin and carboplatin in rats receiving whole body hyperthermia. *Proc. Am. Assoc. Cancer Res.*, **31,** 392.

VON HOFF, D.D., SHILSKY, R., REICHERT, C.M., REDDICK, R.L., ROZENCWEIG, M., YOUNG, R.C. & MUGGIA, F.M. (1979). Toxic effects of cis-dichlorodiammineplatinum (II) in man. *Cancer Treat. Rep.*, **63,** 1527–1531.

WAGSTAFF, A.J., WARD, A., BENFIELD, P. & HEEL, R.C. (1989). Carboplatin: A preliminary review of its pharmacodynamic and pharmacokinetic properties and therapeutic efficacy in the treatment of cancer. *Drugs*, **37,** 162–190.

WILTSHAW, E. (1985). Ovarian trials at the Royal Marsden. *Cancer Treat. Rev.*, **12,** (Suppl. A): 67–71.

WONDERGEM, J., BULGER, R.E., SIDDIK, Z.H., LEYGRAAF, J.W., STREBEL, F.R., ALONSO, M., TRAVIS, E.L. & BULL, J.M.C. (1989). A comparison of thermal enhancement of *cis*-diamminedichloroplatinum (II) induced renal and intestinal toxicities by whole body hyperthermia in the rat. *Int. J. Radiat. Oncol. Biol. Phys.*, **16,** 1551–1556.

WONDERGEM, J., BULGER, R.E., STREBEL, F.R., NEWMAN, R.A., TRAVIS, E.L., STEPHENS, L.C. & BULL, J.M.C. (1988*a*). Effect of *cis*-diamminedichloroplatinum (II) combined with whole body hyperthermia on renal injury. *Cancer Res.*, **48,** 440–446.

WONDERGEM, J., STREBEL, F.R., SIDDIK, Z.H., NEWMAN, R.A. & BULL, J.M.C. (1988*b*). The effect of anaesthetics on cis-platinum-induced toxicity at normal temperatures and during whole-body hyperthermia; the influence of NaCl concentration of the vehicle. *Int. J. Hyperthermia*, **4,** 643–654.

WONDERGEM, J., STEPHENS, L.C., STREBEL, F.R., BABA, H., OHNO, S., SIDDIK, Z.H., NEWMAN, R.A. & BULL, J.M.C. (1991). Effect of adriamycin combined with whole body hyperthermia on tumor and normal tissues. *Cancer Res.*, **51,** 3559–3567.

XU, M.J. & ALBERTS, D.S. (1988). Potentiation of platinum analogue cytoxicity by hyperthermia. *Cancer Chemther. Pharmacol.*, **21,** 191–196.

ZAKRIS, E.L., DEWHIRST, M.W., RIVIERE, J.E., HOOPES, P.J., PAGE, R.L. & OLESON, J.R. (1987). Pharmacokinetics and toxicity of intraperitoneal cisplatin combined with regional hyperthermia. *J. Clin. Oncol.*, **5,** 1613–1620.

ZWELLING, L.A. (1987). Cisplatin and new platinum analogs. In Pinedo, H.M., Longo, D.L., Chabner, B.A. (eds), *Cancer Chemotherapy and Biological Response Modifiers*, pp. 71–80. Amsterdam: Elsevier Science Publishers B.V.